# *Candida auris* emergence in the Himalayan foothills: First case report from Uttarakhand, India

**DOI:** 10.18502/cmm.6.1.2509

**Published:** 2020

**Authors:** Suneeta Meena, Ranjana Rohilla, Neelam Kaistha, Arpana Singh, Pratima Gupta

**Affiliations:** Department of Microbiology, All India Institute of Medical Sciences, Rishikesh, India

**Keywords:** Candidemia, Candida auris, Caspofungin, Fluconazole, Intensive care unit (ICU)

## Abstract

**Background and Purpose:**

Candida auris is a rapidly emerging fungus, which is considered globally a cause of concern for public health. This report describes the first case of *C. auris* fungemia from a tertiary care hospital in the hilly state of Uttarakhand in India.

**Case report:**

The patient was a 37-year-old female who underwent a Whipple procedure for the carcinoma of the head of the pancreas. She developed fever 12 days after the operation while recovering from surgery in the hospital. Blood culture yielded *C. auris* which was identified by the matrix-assisted laser desorption/ionization-time of flight mass spectrometry (Bruker Daltonics, Germany). The patient was successfully treated with caspofungin.

**Conclusion:**

In conclusion, *C. auris* is potentially multidrug resistant, resulting in nosocomial outbreaks and sporadic infections which can be potentially prevented when identified early by implementing contact precautions.

## Introduction

*Candida auris* is an emerging fungus that is a cause of global concern. Healthcare-associated outbreaks and cases have been reported worldwide, and the number of countries involved has increased to 37 [1-3]. In India, the emergence of *C. auris* was noted in sporadic outbreaks and in a multicenter study of candidemia performed in 27 intensive care units (ICUs) in 2011 [4-6]. This is an unpleasant event as *C. auris* is commonly multidrug-resistant with some isolates displaying pan-resistance to the available antifungal drugs. Herein, we report the first case of *C. auris* fungemia in a 37-year-old female in Uttarakhand, India. The female underwent Whipple procedure at a tertiary care hospital in Uttarakhand and was successfully cured with caspofungin.

## Case report

A 37-year-old housemaker was admitted to the Department of General Surgery of the All India Institute of Medical Sciences in Rishikesh, Uttarakhand, in March 2019. She had the complaints of severe constricting pain in the last 12 days. The pain was aggravated with the intake of solid food and relieved after the administration of medications. She had a history of yellowish discoloration of the eyes and urine in the past 10 days. On physical examination, the abdomen was soft and non-tender. No other abnormality was detected on auscultation and digital rectal examination. Examination of other systems was also within the normal limits.

Laboratory workup revealed cholestasis (bilirubin of 11.91 mg/dL, alkaline phosphatase of 320 U/L, and gamma-glutamyl transferase of 627 U/L) with elevated liver enzymes (aspartate aminotransferase of 479 U/L and alanine aminotransferase of 330 U/L). In addition, the contrast-enhanced computed tomography of the abdomen and pelvis showed features suggestive of the carcinoma of the head of the pancreas. Carbohydrate antigen 19-9 level was elevated to 84.4 U/mL. 

The patient was planned for Whipple procedure; therefore, all pre-operative evaluations were performed. After obtaining written informed consent, the patient underwent surgery under general anaesthesia. The surgery lasted for around 10 hours. Intraoperative period was uneventful, except for internal jugular vein thrombosis while attempting to insert central line. After the procedure, the patient was transferred to the ICU and kept on ventilatory support. The patient was started on total parenteral nutrition and broad-spectrum intravenous antibiotics (i.e., piperacillin/tazobactam [3.375 g] within 6-hour intervals, amikacin [9 g] daily, and metrogyl [7.5 mg/kg]). The patient was extubated 2 days after the operation and then maintained on non-invasive ventilation and transferred to the surgery ward. 

**Figure 1:**
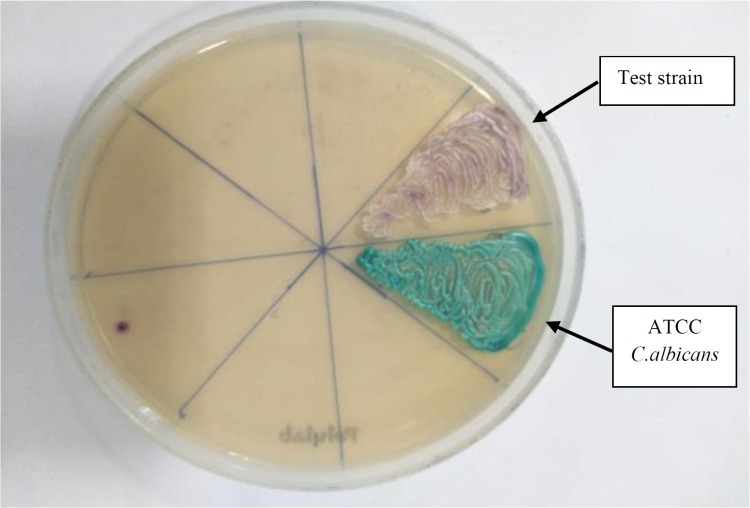
CHROM agar (HiMedia) showing colony color of isolate (test) after 48 hours of aerobic incubation

The vital signs were stable, and urine output was maintained. On day 7 post-operation, right-sided drainage and foleys catheter were removed. In addition, 10 days after the surgery, the patient underwent pigtail insertion for intraabdominal fluid collection. On day 12, she developed fever, back pain, blood urine; therefore, central line tip culture was sent. The patient was started on cefoperazone/sulbactam, amikacin, and metronidazole. Fourteen days post-surgery, the patient initiated on a soft oral diet and also fluconazole was added to the treatment regimen. Episodes of fever still continued. 

On day 15, blood culture reports revealed the presence of *Candida auris*. Blood culture was performed by means of the automated BACT/ALERT 3D Microbial Identification system (Biomerieux, France) which signaled a positive growth after 3 days of incubation. On subculture, tiny white opaque, non-hemolytic colonies were obtained on 5% sheep blood agar. Gram staining revealed Gram-positive budding yeast cells. Germ tube test was performed using *C. albicans *ATCC 90028 as control and demonstrated negative results for germ tube production. 

The colony was subcultured on CHROM agar (HiMedia, India), which revealed colorless colonies in 24 h which became pink-purplish on further incubation [Figure 1]. The yeast was identified as *C. auris* by the matrix-assisted laser desorption/ionization-time of flight mass spectrometry (MALDI-TOF MS; Bruker Daltonics, Germany) with a score of 1.8 with direct formic acid extraction. Thereafter, the patient was started on caspofungin (50 mg/day). She showed improvement symptomatically, her fever subsided, and she could tolerate oral feeds well.

Caspofungin was continued for 2 weeks and the result of repeat blood culture was negative. Caspofungin was continued for a week after discharge. The patient was followed up a week later and recovered completely. Therefore, caspofungin was stopped. On the follow-up performed a month later, the patient recovered completely.

To confirm the identity of the isolate, internal transcribed spacer (ITS) regions (ITS1-5.8S-ITS2) were amplified using ITS1 (5′-TCCGTAGGTGAACCTGCGG-3) and ITS4 (5′-TCCTCCGCTTATTGATATGC-3′) as described previously [7]. Comparison of the nucleotide sequence of our isolate with the Gene Bank database using the Basic Local Alignment Search Tool (BLAST) algorithm yielded 100% homology with the ex-type strain of *C.auris* (accession no. MN796100). 

**Figure 2.**
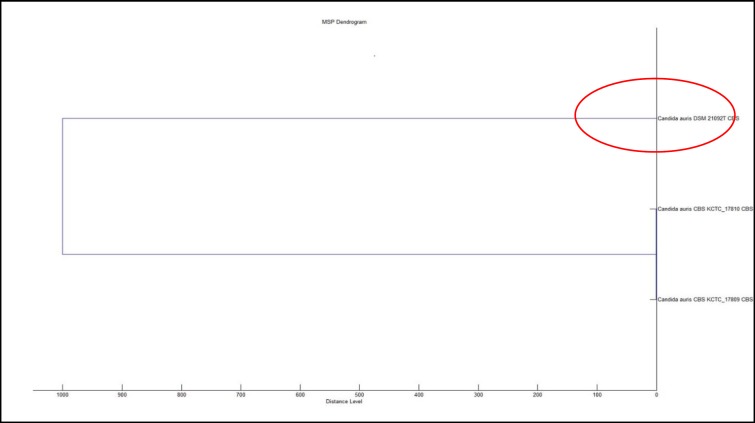
Bruker Biotyper MSP dendrogram with red circle showing the strain examined in this study

Antifungal susceptibility was performed on Vitek 2 (BioMérieux Craponne, France). It was found sensitive to caspofungin (MIC≤0.25), micafungin (MIC≤0.06), and voriconazole (MIC≤0.5), intermediately sensitive to flucytosine (MIC 8), and resistant to fluconazole (MIC≥64) and amphotericin B (MIC≥16). The dendrogram was generated using the respective functionality of the MALDI-TOF MS Biotyper 3.1 offline client [Figure 2].

## Discussion

Concerning the epidemiology of candidemia, a large multicenter observational study investigating ICU-acquired candidemia in India found an overall incidence of 6.51 cases per 1,000 ICU admissions [5]. In another similar study, the prevalence of *C. auris* candidemia was reported as 5.3% [6]. This species has also been recovered from cutaneous infections and otomycosis [1]. So far, this worrisome bug has not been reported from the hilly part of India, investigated in the current study. To the best of our knowledge, this is the first case from the state of Uttarakhand in India. 

*Candida auris* has been isolated in many countries across the world, and whole-genome sequencing has revealed five distinct clades, namely South Asia (India), South Africa, South America, East Asia, and the Middle East [8,9]. This is the first and only case of *C. auris* out of 36 yeasts having been isolated from blood at our institute in the last 6 months. We routinely identify all clinically significant fungal pathogens by MALDI-TOF MS. There was no other patient besides our case in the mentioned ICU. The patient was located in the corner of the ward. In addition, contact precautions were followed strictly. 

Sampling from other sites of the body of the patient did not yield any significant findings. Although all yeasts are identified at the species level in our laboratory, we cannot exclude the possibility that a *C. auris* isolate might have gone undetected in the presence of mixed culture in the urine or pus specimens. Blood stream infection in this particular patient appeared to be healthcare-associated. It could have been facilitated by such risk factors as prolonged surgery, central venous catheter, broad-spectrum antibiotics, fluconazole prophylaxis, and prolonged hospital stay.

Our isolate exhibited higher MIC for fluconazole and amphotericin B which is in concordance with the reports in the literature [1, 5]. Our case was successfully managed with caspofungin since it could be correctly identified by the MALDI-TOF MS. In conclusion, *C. auris* as a potentially multidrug-resistant agent associated with nosocomial outbreaks and sporadic infections can be potentially prevented when identified early by implementing contact precautions.

## Conclusion

In conclusion, *C. auris* is potentially multidrug resistant, resulting in nosocomial outbreaks and sporadic infections which can be potentially prevented when identified early by implementing contact precautions.

## References

[1] Abastabar M, Haghani I, Ahangarkani F, Rezai MS, Taghizadeh Armaki M, RoodgariS (2019). Candida auris otomycosis in Iran and review of recent literature. Mycoses.

[2] Vogelzang EH, Weersink AJ, van Mansfeld R, Chow NA, Meis JF, van Dijk K (2019). The first two cases of Candida auris in the Netherlands. J Fungi (Basel).

[3] ( 2019). Tracking Candida auris.

[4] Chowdhary A, Voss A, Meis JF (2016). Multidrug-resistant Candida auris: new kid on the block in hospital associated infections?. J Hosp Infect.

[5] Chakrabarti A, Sood P, Rudramurthy SM, Chen S, Kaur H, Capoor M (2015). Incidence, characteristics and outcome of ICU-acquired candidemia in India. Intensive Care Med.

[6] Rudramurthy SM, Chakrabarti A, Paul RA, Sood P, Kaur H, Capoor MR (2017). Candida auris candidaemia in Indian ICUs: analysis of risk factors. J Antimicrob Chemother.

[7] White TJ, Bruns TD, Lee SB, Taylor JW, Innis N, Gelfand D (1990). PCR-protocols and applications-a laboratory manual. New York: Academic Press.

[8] Chow NA, de Groot T, Badali H, Abastabar M, Chiller TM, Meis JF (2019). Potential fifth clade of Candida auris, Iran, 2018. Emerg Infect Dis.

[9] Lockhart SR, Etienne KA, Vallabhaneni S, Farooqi J, Chowdhary A, Govender NP (2017). Simultaneous emergence of multidrug-resistant Candida auris on 3 continents confirmed by whole-genome sequencing and epidemiological analyses. Clin Infect Dis.

